# Pregnancy Outcomes After Transcervical Radiofrequency Ablation of Uterine Fibroids with the Sonata System

**DOI:** 10.1089/gyn.2021.0136

**Published:** 2022-06-13

**Authors:** Ladina Christoffel, Ralf Bends, David Toub, Sven Schiermeier, Gregor Pschadka, Matthias Engelhardt, Stephen Quinn, Michael Hartmann, Marwan Habiba, Ricardo Felberbaum, Anke Brössner, Cordula Schippert, Thomas Römer

**Affiliations:** ^1^ Chefärztin Gynäkologie/Geburtshilfe, Spital Oberengadin, Samedan, Switzerland.; ^2^ Evangelisches Klinikum Köln-Weyertal, Köln, Germany.; ^3^ Gynesonics, Redwood City, California, USA.; ^4^ Zentrum für Frauenheilkunde und Geburtshilfe, Marien Hospital, Witten, Germany.; ^5^ Gynäkologie, Josephs-Hospital Warendorf, Warendorf, Germany.; ^6^ Department of Gynaecology, St Mary's Hospital, Imperial College Healthcare Trust, London, United Kingdom.; ^7^ Marienkrankenhaus Schwerte, Schwerte, Germany.; ^8^ Obstetrics and Gynaecology, University Hospitals of Leicester NHS Trust, Leicester, United Kingdom.; ^9^ Klinikverbund-Allgäu, Kempten, Germany.; ^10^ Medizinische Hochschule Hannover, Hannover, Germany.

**Keywords:** uterine fibroid, leiomyoma, radiofrequency ablation, Sonata System, transcervical fibroid ablation

## Abstract

**Objective:**

To describe pregnancy outcomes in women who conceived after undergoing transcervical fibroid ablation (TFA) as treatment for symptomatic uterine fibroids.

**Materials and Methods:**

TFA was used to treat symptomatic uterine fibroids with radiofrequency energy, both under clinical trial protocol and commercial usage in hospitals in Europe, the United Kingdom, Mexico, and the United States. All women who reported pregnancies to their physicians after undergoing TFA with the Sonata^®^ System and provided consent for use of their data were included.

**Results:**

There have been 36 pregnancies representing 20 deliveries among 28 women who were treated with TFA. Five women conceived more than once postablation, and four conceived as a result of assisted reproductive technology (ART). Outcomes include 8 vaginal deliveries, 12 Cesarean sections, 3 therapeutic abortions, and 8 first trimester spontaneous abortions (four occurring in a patient with a history of recurrent pregnancy loss and an immunologic disorder). Five women are currently pregnant, two of whom previously delivered after TFA. There were no 5-minute Apgar scores <7, and all neonates weighed >2500 g. All deliveries occurred at ≥37 weeks except for one delivery at 35 6/7 weeks. There were no uterine ruptures or abnormal placentation and no reports of postpartum hemorrhage or stillbirths. Ablated fibroids included transmural, submucous, and intramural myomata up to 7 cm in diameter.

**Conclusions:**

Normal pregnancy outcomes at term have occurred after TFA with the Sonata System, including in women with recurrent abortion and in those undergoing ART. There were no instances of low Apgar scores, low birthweight, stillbirth, postpartum hemorrhage, or uterine rupture (FAST-EU, NCT01226290; SONATA, NCT02228174; SAGE, NCT03 118037). (J GYNECOL SURG 38:207)

## Introduction

Uterine fibroids are highly prevalent benign lesions within the myometrium, and are present in ∼70% of white women and >80% of black women in the United States.^1^ As the most common indication for hysterectomy in the United States, fibroids are associated with significant costs to the health care system of as much as $34.4 billion/year, including direct costs of $4.1–9.4 billion.^2^ Symptomatic uterine fibroids have a negative impact on a woman's quality of life, due to their association with heavy menstrual bleeding (HMB), bulk symptomatology, dyspareunia, and other problems in up to 50% of women in the United States.^3^

Hyperthermic ablation of uterine fibroids using magnetic resonance-guided focused ultrasound (MRgFUS) and radiofrequency (RF) energy has proven safe and effective in women who do not desire hysterectomy.^4–16^ In particular, transcervical fibroid ablation (TFA) is an incisionless procedure involving the delivery of RF energy to targeted fibroids and is performed with the Sonata^®^ System (Gynesonics, Inc., Redwood City, CA, USA). Sonata enables the transcervical ablation of uterine fibroids under real-time integrated intrauterine sonography, and multiyear outcomes have been described in detail.^14,15,17,18^ Of note, TFA with the Sonata System has been associated with significant and durable improvements in menstrual bleeding and overall quality of life, along with a low 3-year reintervention rate of 8.2%, high patient satisfaction, and confirmed safety.^15^ A recent systematic review noted “Radiofrequency ablation with the Sonata® System represents a minimally invasive, organ-preserving treatment option in patients with symptomatic uterine myomas, associated with clinically meaningful improvement of myoma related symptoms.”^8^ Because of its integrated intrauterine ultrasound probe, the Sonata System can ablate a wider repertoire of fibroids than operative hysteroscopy, including intramural, transmural, and nonpedunculated subserous fibroids, in addition to nonpedunculated submucous myomata. Multiple ablations are utilized to ablate fibroids ≥5 cm as needed, and treatment of such large fibroids with TFA has been demonstrated to be effective in improving abnormal uterine bleeding, overall fibroid symptoms and quality of life.^19^

The Sonata System has been cleared by the US Food & Drug Administration (FDA) and received the Conformitè Europëenne (CE) Mark in the European Union, and the FDA labeling notes that as a uterus-conserving alternative to hysterectomy, treatment with the Sonata System does not eliminate the possibility of pregnancy. A recently updated ACOG Practice Bulletin notes that TFA is “similarly effective” to laparoscopic and transvaginal RF ablation of uterine fibroids with regard to reduction in fibroid volume and improvement in quality of life.^20^ A 2021 review of fibroid treatment options by a panel of expert German, Swiss, and Austrian gynecologists provided a consensus opinion that TFA is preferred for the treatment of International Federation of Gynecology and Obstetrics (FIGO) type 2, type 3, type 4, and type 2–5 fibroids, and for all fibroids that are difficult to access using surgical treatment.^21^

Of the hyperthermic fibroid ablation modalities, MRgFUS has been approved by the FDA for use in women who desire fertility.^12^ Pregnancy outcomes after focused ultrasound (FUS) have been favorably compared with those after laparoscopic myomectomy.^22^ There have also been reports of successful pregnancy outcomes after RF ablation of uterine fibroids, including TFA.^23–29^ Of note, three published clinical studies involving TFA have collected data regarding pregnancy.^10,15,30^ Two of these (FAST-EU and SONATA) were prospective clinical trials that enrolled a total of 197 women, whereas SAGE was a postmarket global registry that included women with ablated fibroids ranging from <1 cm in maximum diameter to >10 cm. There were patients in all three studies who reported pregnancies post-TFA. In addition, women who have been treated in Europe under commercial (nonprotocol) usage of the Sonata System have also reported pregnancies after TFA.

This article reports the cumulative global experience with pregnancy after TFA with the Sonata System, both within clinical studies (FAST-EU, SONATA, and SAGE) and during commercial use.

## Materials and Methods

Women with symptomatic uterine fibroids were treated with the Sonata System either under clinical trial protocols (if enrolled in FAST-EU, SONATA, or SAGE) or under postmarket commercial usage guided by the manufacturer's instructions for use. Clinical trial protocols for the three trials cited in this article are posted at ClinicalTrials.gov (FAST-EU, NCT01226290; SONATA, NCT02228174; SAGE, NCT03118037). The FAST-EU clinical trial was a 12-month prospective study that commenced in January 2011 and was completed in March 2014. This trial enrolled 50 women treated at 7 sites in Europe (6 sites) and Mexico (1 site). The SONATA clinical trial was a 36-month prospective trial that enrolled 147 women at 22 sites in the United States (21 sites) and Mexico (1 site). SONATA commenced enrollment in April 2015 and enrollment was completed in October 2016. SAGE is an ongoing postmarket global clinical registry that began in April 2017 and has enrolled 160 women to date at 7 sites in Europe with a mean follow-up of 5.3 months and an upper range of 25 months.^10,17,30^

For FAST-EU, women were enrolled if they had symptomatic uterine fibroids associated with HMB and at least one indenting fibroid as per the FIGO subclassification (FIGO type 1, type 2, or type 2–5); patients were followed for 12 months. In the SONATA clinical trial, women were required to have at least one indenting or abutting fibroid (type 1, type 2, type 2–5, and type 3). In SAGE, patients were enrolled if they had selected TFA to treat their fibroid symptoms and were willing to participate in the study.

In each trial, patients provided written informed consent and Institutional Review Boards/Ethics Committees at each participating center provided study protocol approval. Each clinical trial was performed in accordance with the ethical standards as laid down in the 1964 Declaration of Helsinki and its later amendments. Women who were treated with the Sonata System commercially (postmarket) provided their informed consent to undergo elective treatment for symptomatic fibroids.

Data regarding women who conceived and delivered while enrolled in FAST-EU, SONATA, or SAGE were provided by the clinical study sites and governed by their respective Ethics Committees and Institutional Review Boards and informed consent requirements. Pregnancy data for women who conceived outside of a clinical study were collected by their individual treating physicians and each woman individually provided her informed consent regarding inclusion of their anonymous pregnancy data within this report. Anonymized data were collected by each treating physician regarding patient demographics, fibroid size and FIGO type, TFA procedure date, and pregnancy outcomes (pregnancy outcome, delivery route, estimated gestational age, birth weight, Apgar scores, and complications). All data regarding pregnancy outcomes were collected in a flat file database using Microsoft Excel (Microsoft, Redmond, WA, USA) and were analyzed descriptively. Duplicative data (e.g., fibroid size and FIGO type) for patients who delivered more than once after TFA were excluded. Demographic and fibroid data for three patients whose first pregnancies post-TFA are ongoing are not provided as consent for use of their data is pending until their pregnancy outcomes have been realized.

## Results

Since June 2011 when the first patient was treated with TFA as part of the FAST-EU clinical trial, there have been 36 pregnancies among 28 women. This represents 20 deliveries, 3 therapeutic abortions, and 8 first-trimester spontaneous abortions (SAbs) with 5 ongoing pregnancies, 2 of which are in patients who had prior deliveries after TFA. Patient and fibroid characteristics are summarized in Table 1; delivery outcomes are summarized in Table 2. As noted, this excludes three women with ongoing pregnancies who had not yet provided consent for inclusion of their individual data. Nine women were nulligravidae at the time of their TFA procedures. Thirteen deliveries (65%) among 13 unique patients took place after postmarket use of the Sonata System, whereas 7 deliveries (35.0%) occurred among 7 individual women who were part of a clinical trial. Ethnicity information about each patient was not available from the individual treating physicians.

**Table 1.** Patient and Ablated Fibroid Characteristics

Women (*n*)	28
Age at the beginning of each post-TFA pregnancy (years)^a^	
Mean ± SD	35.6 ± 5.0
Median (range)	37 (22, 45)
Gravidity^a,b^	
Mean ± SD	1.5 ± 1.3
Median (range)	1 (0, 5)
Parity^a,b^	
Mean ± SD	0.5 ± 0.7
Median (range)	0 (0, 2)
Nulligravidae (*n*)^a^	9
Clinical trial patients (*n*)	9
FAST-EU	1
SONATA	2
SAGE	6
Patients treated postmarket (*n*)	19
Total no. of fibroids ablated (*n*)^a^	35
Ablated fibroid diameter (cm)^a^	
Mean	3.5 ± 1.7
Median (range)	3.1 (1, 7)
No. of fibroids ablated/patient^a^	
Mean	1.4 ± 1.1
Median (range)	1 (1, 6)
Ablated fibroid FIGO type, *n* (%)^a^	
Type 1	1 (2.9)
Type 2	5 (14.3)
Type 3	3 (8.6)
Type 4	8 (22.9)
Type 5	2 (5.7)
Type 6	0 (0)
Type 2–5	16 (45.7)

^a^ Exclusive of three women with ongoing pregnancies after postmarket use of the Sonata System whose consent for inclusion of their anonymous information is pending until their pregnancy outcomes have been realized (total number of post-TFA pregnancies = 36).

^b^ Gravidity and parity are all considered before each post-TFA pregnancy.

ART, assisted reproductive technology; FIGO, International Federation of Gynecology and Obstetrics; ICSI, intracytoplasmic sperm injection; IVF, *in vitro* fertilization; SD, standard deviation; TFA, transcervical fibroid ablation.

**Table 2.** Pregnancies and Outcomes

Total no. of pregnancies	36
Completed (*n* = 25 women), *n* (%)	31 (86.1)
Ongoing, *n* (%)	5 (14.3)
Patients without a prior post-TFA delivery	3
Patients with a prior post-TFA delivery	2
Patients with >1 pregnancy after TFA (*n*)	5
Patients conceiving via ART	4
IVF	2
ICSI	2
Deliveries, *n* (%)	20 (64.5)^a^
Clinical trial	7 (35.0)^b^
Postmarket	13 (65.0)^b^
Vaginal	8 (40.0)^b^
Cesarean section	12 (60.0)^b^
1°	9 (75.0)
Repeat	3 (25.0)
Abortions, *n* (%)	11 (35.4)^a^
Tab	3 (9.7)^a^
Sab	8 (25.8)^a^
First trimester	8 (100)
Second trimester	0 (0)
Preterm delivery <37 weeks, *n* (%)	1 (5.0)^b^
Postpartum hemorrhage, *n* (%)	0 (0)
Five-minute Apgar score <7, *n* (%)	0 (0)
Birthweight <2500 g, *n* (%)	0 (0)
Birthweight (g)	
Mean ± SD	3456.0 ± 480.3
Median (range)	3400 (2555, 4500)

^a^ Denominator = completed pregnancies (*n* = 31).

^b^ Denominator = deliveries (*n* = 20).

ART, assisted reproductive technology; SAb, spontaneous abortion; SD, standard deviation; TAb, therapeutic (elective) abortion; TFA, transcervical fibroid ablation.

Thirty-five fibroids were ablated among 25 women. On average, 1.4 ± 1.1 fibroids were ablated per patient (median 1.0; range 1–6), and the mean ablated fibroid diameter was 3.5 ± 1.7 cm (median 3.1 cm; range 1.0–7.0 cm). Transmural fibroids were the most common ablated fibroid type (FIGO type 2–5), but type 1, type 2, type 3, type 4, and type 5 fibroids were also ablated.

Five women conceived more than once postablation, and four women conceived as a result of assisted reproductive technology (ART). Assisted reproduction included *in vitro* fertilization (*n* = 2) and intracytoplasmic sperm injection (*n* = 2). Although time from TFA to conception was not reported for most of the patients, the earliest documented conception occurred in a patient in the FAST-EU trial who conceived ∼3.5 months after the procedure.^24^

Of the eight first-trimester SAbs that occurred among five women, four of these occurred in an individual with a history of recurrent pregnancy loss and an immune disorder. This patient originally underwent TFA in August 2017 after a single pregnancy loss at 14 weeks believed at that time to be secondary to a 3.9-cm FIGO type 2–5 (transmural) fibroid. Beginning 1 year later, she experienced four additional first trimester pregnancy losses and was diagnosed with an “immunological problem.” The presence of antiphospholipid antibodies was believed by the reporting physician to be the causative factor. The patient conceived again in early 2020, and her pregnancy was uncomplicated until term when she developed HELLP syndrome, underwent a trial of labor followed by an emergency Cesarean section due to nonreassuring fetal monitoring. Umbilical artery pH was 7.00 and Apgar scores were 9 and 10 at 5 and 10 minutes, respectively. The SAbs are detailed in Table 3.

**Table 3.** Details of First Trimester Pregnancy Losses After Transcervical Fibroid Ablation

Case	Age at loss	Gravidity^a^	Parity^a^	No. of ablated fibroids	Maximum fibroid diameter (cm)	FIGO type	Comments
1	40	1	1	1	4.8	2	Occurred 29 months post-TFA
2	40	0	0	2	5.4 1	2–5 5	Occurred 22 months post-TFA
3	45	2	0	1	1.3	2	IVF pregnancy; SAb occurred 38 months post-TFA
4	31	0	0	1	3.4	2–5	Occurred 8 months post-TFA
5	36	1	0	1	3.9	2–5	“Immune disorder”
6	36	2	0	1	3.9	2–5	Same patient as case #5; “immune disorder”
7	37	3	0	1	3.9	2–5	Same patient as case #5 “immune disorder”
8	37	4	0	1	3.9	2–5	Same patient as case #5; “immune disorder”

^a^ Gravidity and parity are all considered before each post-TFA pregnancy.

There were no low (<7) 5-minute Apgar scores reported, and birth weights were within normal limits (>2500 g). All deliveries were at term (≥37 weeks) except for one delivery at 35 6/7 week's gestation (5.0% of all deliveries). No deliveries were associated with stillbirth, uterine rupture, fetal growth restriction, or postpartum hemorrhage. There were no placentation abnormalities noted by the delivering obstetricians nor any notations of necrotic fibroid remnants. Reported pregnancy complications consisted of fetal macrosomia (three patients), hemolysis with elevated liver enzymes and a low platelet count (HELLP syndrome), premature rupture of membranes with meconium-stained amniotic fluid, breech presentation and pyelonephritis at 16 weeks' gestation.

Cesarean section was performed in 12 women (60.0% of deliveries). Of the 12 Cesarean sections, 9 (75.0%) were primary and 3 (25.0%) were repeat Cesarean sections. Detailed information is provided in Table 4. In all cases, these operative deliveries were performed for obstetric indications, such as nonreassuring fetal monitoring or previous multiple Cesarean sections or were elective upon patient request.

**Table 4.** Details of Cesarean Sections (*n* = 11 Women) After Treatment with Transcervical Fibroid Ablation

Index	Type of C/S	Gestational age (weeks)	Indication
1	Primary	38 6/7	Patient preference
2	Primary	38 2/7	Fetal macrosomia (4500 g)
3	Primary	40 5/7	Nonreassuring fetal tracing, failed TOL, HELLP syndrome
4	Primary	41 3/7	FTP
5	Primary	38 5/7	Fetal macrosomia (4040 g)
6	Primary	39 1/7	Breech presentation
7	Primary	39 6/7	Nonreassuring fetal tracing, PROM, MSAF
8	Primary	39 1/7	Patient preference
9	Repeat	38 1/7	Elective
10	Repeat	38 2/7	Elective, fetal macrosomia (4005 g)
11	Repeat	38 1/7	Elective
12	Primary	39 4/7	FTP, nonreassuring fetal tracing

FTP, failure to progress; HELLP, hemolysis with elevated liver enzymes and a low platelet count; MSAF, meconium-stained amniotic fluid; PROM, prolonged rupture of membranes; TOL, trial of labor.

## Discussion

TFA with the Sonata System has been associated with uncomplicated vaginal and operative deliveries at term.^10,14,23–25^ The current data in totality, as presented in this article, provide additional evidence that hyperthermic ablation with RF energy may be compatible with a desire for future fertility.

Initial clinical trials of MRgFUS, laparoscopic fibroid ablation (LFA), and TFA excluded women who expressed a desire for future pregnancy, which has generally limited the published clinical experience with pregnancy outcomes after hyperthermic fibroid ablation to case reports and case series. Nonetheless, there has been growing evidence that hyperthermic fibroid ablation, whether with FUS or RF energy, is an appropriate consideration for women who desire pregnancy in the future. Keltz et al. performed a systematic review of pregnancies after hyperthermic fibroid ablation, including TFA.^29^ The review, which included 102 pregnancies after FUS and 20 after either TFA or LFA, noted favorable pregnancy outcomes and a lack of uterine rupture after TFA, LFA, and FUS. Indeed, the US FDA has approved an indication for the use of MRgFUS in women who desire future pregnancy.^12^ Pregnancy outcomes after LFA have also been favorable, with 30 pregnancies (26 deliveries, 4 SAbs) reported among 28 women.^28^

The 36 post-TFA pregnancies reported herein bring the total number of reported pregnancies after either TFA or LFA to 66. Regarding TFA, there have been no reports of uterine ruptures, stillbirths or abnormal placentation. The SAb rate (8 of 31 completed pregnancies; 25.8%) reflects the inclusion of four losses from a single patient and attributed to antiphospholipid syndrome. The SAb rate would have been 14.8% (4 of 27 completed pregnancies) if her four post-TFA miscarriages were excluded as outliers. Regardless, it is known that first-trimester pregnancy losses are common, especially among multiparas, with 43% of parous women having at least one first trimester SAb.^31^ Nulliparas were found to have a SAb rate of 14% in the general population, and this rises to 26% after one delivery. Age is another important factor, and SAb rates ≥50% are associated with maternal age ≥35 years.^31^ As half of the women in our overall cohort were >37 years at the start of their pregnancies, the SAb rate of 25.8% does not exceed normal limits. It should be noted that three of the five women who experienced a SAb after TFA were at least 40 years old. The SAb rate for TFA is also slightly less than the 28% SAb rate reported by Rabinovici et al. in their series of 51 pregnancies after MRgFUS.^32^

The 12 Cesarean sections reported after TFA represent a 60.0% rate of abdominal delivery, and 75% (9/12) of these were primary Cesarean sections. Two of the nine primary Cesarean sections reflected patient preference, whereas the others were for standard obstetrical indications (fetal macrosomia, dystocia, etc.) unrelated to prior fibroid ablation. The 60.0% abdominal delivery rate is similar to that reported by Berman et al. (50%) after LFA.^28^ The abdominal delivery rate after MRgFUS was 36% as reported by Rabinovici et al.^32^

Of note, there were no reports of abnormal placentation or postpartum hemorrhage for pregnancies after TFA. Such complications appear uncommon after hyperthermic fibroid ablation in general, whereas they have been more frequently associated with pregnancies after uterine artery embolization.^33–35^

This case series has several strengths. Most patients (19/28; 67.9%) were treated on a postmarket basis, representing real-world data. The majority (25/35; 71.4%) of treated fibroids either indented or abutted (FIGO type 3) the endometrial cavity; such fibroids are believed to have a negative impact on achieving pregnancy, although the association between myomata and fertility remains unclear.^36–38^

There are also a few limitations. Outside of nine patients who had enrolled in clinical trials of TFA, this case report largely represents women whose pregnancies and pregnancy outcomes were voluntarily reported by their treating physicians. As such, the number of pregnancies could be underrepresented (even in the clinical trial cohort). Some pregnancies at outside institutions may not have been reported to the physicians responsible for the antecedent TFA procedure. This case series cannot directly address the optimal timing of pregnancy after TFA nor the need for abdominal delivery. It is apparent from the FAST-EU clinical trial of TFA that fibroid volume is significantly reduced by 3 months postablation, and for fibroids that indent or abut the endometrial cavity, restoration of a normal cavity would seem optimal for an implanting oocyte.^10,39,40^ Nonetheless, although at least one patient conceived within the first 3.5 months postablation, it is not yet clear if earlier, or later, conception is optimal after TFA. Regarding Cesarean section, although the majority of deliveries in this series were abdominal, they were performed for general obstetric indications such as failure to progress or macrosomia, and 40.0% of deliveries were vaginal with no uterine ruptures occurring. Although it is not yet possible to issue clear guidance as to the preferred route(s) of delivery, there were eight vaginal deliveries in this case series, indicating that normal spontaneous vaginal delivery is possible outside of standard obstetric indications such as macrosomia, malpresentation, and dystocia.

Besides published case reports and case series of pregnancies after TFA, there is additional evidence to suggest that TFA may be compatible with pregnancy. The OPEN clinical trial has demonstrated a minimal risk of intrauterine adhesions after TFA, and the INTEGRITY trial also suggests no material impact of TFA on the integrity of the uterine wall.^41,42^ At the same time, although this case series documents the possibility of safe pregnancy and delivery after TFA, it is not known if TFA has any direct effect, positive or negative, on fertility and pregnancy outcomes. It will be useful to consider the experience of the treatment centers represented in this report as gynecologists and their patients strive to make informed decisions regarding treatment of symptomatic fibroids with the Sonata System.

## Conclusions

Normal pregnancy outcomes at term have occurred after TFA with the Sonata System, including in women with recurrent pregnancy loss and in those undergoing ART. There were no instances of low Apgar scores, low birthweight, stillbirth, postpartum hemorrhage, or uterine rupture.
